# Condylar position changes and prognosis in patients with unilateral mandibular condylar fracture treated non-surgically

**DOI:** 10.1186/s40902-024-00454-5

**Published:** 2024-12-27

**Authors:** Jihye Lim, Woomin Jo, Hyelynn Jeon, Seung Il Song, Jeong Keun Lee

**Affiliations:** 1 Department of Oral and Maxillofacial Surgery, Institute of Oral Health Science, Ajou University School of Medicine, Suwon, Republic of Korea; 2 Office of Biostatistics, Medical Research Collaborating Center, Ajou Research Institute for Innovative Medicine, Ajou University Medical Center, Suwon, Republic of Korea

**Keywords:** Mandibular condylar fracture, Non-surgical treatment, Positional recovery, Prognosis

## Abstract

**Background:**

Non-surgical method is a treatment option for mandibular condylar fracture; however, it is questionable whether bone fragments are adequately reduced and remodeled. The purpose of this study was to identify three-dimensional positional changes in the mandibular condyles in patients treated non-surgically, analyze factors influencing the extent of positional changes, and evaluate clinical prognosis.

**Methods:**

This retrospective study included 31 patients with unilateral mandibular condylar fractures treated non-surgically at the Ajou University Dental Hospital between 2005 and 2023. Computed tomography was performed at the time of the fracture (*T*_0_) and > 6 months after non-surgical treatment (*T*_1_). The extent of recovery of the highest point of the condyle head was measured in three-dimensional *x*-, *y*-, and *z*-axes. At the last follow-up > 6 months after remodeling (*T*_1_), the prognosis was evaluated by clinical examination of mouth opening limitation, malocclusion, deviation on opening, temporomandibular joint disorder (TMD), and facial asymmetry.

**Results:**

Position differences were statistically significant between *T*_0_ and *T*_1_ (paired Student’s *t*-test, *P* < 0.05), and between the *x*-, *y*-, and *z*-axes (Welch’s ANOVA, *P* < 0.05). The degree of positional recovery in the superior and lateral directions showed a statistically significant negative correlation with age (Pearson’s correlation analysis, *P* < 0.05). The average amount of recovery between two age groups of over and under 19 years old was statistically significant (independent *t*-test, *P* < 0.05). Complications included TMD (6.4%), malocclusion (3.2%) and facial asymmetry (3.2%).

**Conclusion:**

After non-surgical treatment, the condyle head of the fractured mandible recovered significantly laterally and superiorly in under 19-year-olds. The functional prognosis was favorable in all age groups. Non-surgical treatment can be an applicable treatment option for patients with mandibular condylar fractures.

## Background

The mandibular condyle is a common fracture site, accounting for 19–52% of mandibular fractures [1]. The condyle is a component of the temporomandibular joint (TMJ) that acts as a buffer against external forces acting on the skull during trauma. Its anatomical positioning leads to common fractures of the condylar process.

However, there is currently no gold standard treatment for mandibular condylar fractures. For decades, there have been debates regarding the treatment of condylar fractures using surgical and non-surgical methods [2–4]. The basic treatment principles for bone fractures include open reduction and internal fixation using plates and screws. A prerequisite for functional recovery is repositioning bone fragments and restoring continuity as much as possible before the fracture. On the other hand, closed reduction is also a treatment option for mandibular condylar fracture. The maxilla and mandible are fixed using various methods, such as arch bar, screws, eyelet wiring, and circummandibular wiring. During stable immobilization, which provides an environment for bone union between fragments, bone remodeling of the condyle is observed after non-surgical treatment.

Nonetheless, it is questionable whether bone fragments are adequately reduced and remodeled after closed reduction, which influences clinical functional prognosis. Some fractured condyles nearly return to their original position, whereas others remain significantly displaced after the bone union. There are not enough studies concerning three-dimensional positional changes of the condyle head and factors involved in remodeling. In addition, computed tomography images could provide three-dimensional differences in movements due to accurate images without overlap with adjacent anatomical structures compared with plane radiographs such as panoramic views.

This study aimed to identify three-dimensional positional changes of mandibular condyles in patients treated non-surgically, analyze the factors influencing the extent of positional changes, and evaluate clinical prognosis.

## Patients and methods

### Patients

**Fig. 1:**
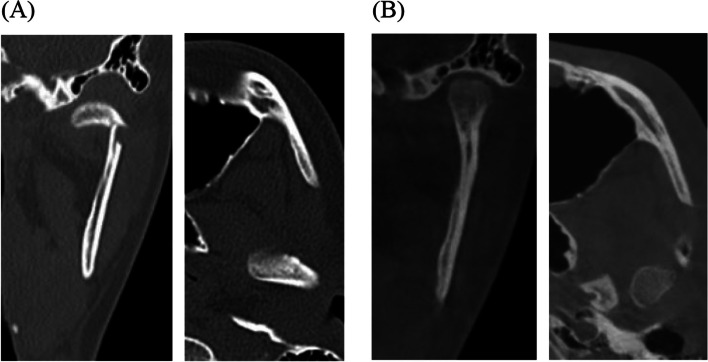
A case of mandibular condylar fracture and its recovery after treatment with closed reduction. A 14-year-old female patient showed favorable positional recovery after closed reduction. **A** Coronal and axial views of the left condyle at the time of fracture were shown. **B** Coronal and axial views of the left condyle after 2.5 years of remodeling were shown

This study included 31 patients who visited the Department of Oral and Maxillofacial Surgery at Ajou University Dental Hospital between 2005 and 2023 for unilateral mandibular condylar fractures and who were treated non-surgically. All patients underwent computed tomography (CT) and clinical examination after > 6 months of follow-up (Fig. 1). After reviewing 331 patients with condylar fractures, subjects who were observed for < 6 months and followed up without a CT scan, patients with bilateral mandibular condylar fractures, and patients without deviation or displacement of bone segment were excluded. Study participant characteristics are shown in Table 1. This study was approved by the Institutional Review Board (IRB) of Ajou University Hospital (AJOUIRB-DB-2024–208), which waived the requirement for informed consent.

**Table 1:** Patients’ characteristics

Variable	*n* = 31
**Age (years)**	
**Mean (SD)**	26.55 (18.39)
**<** **19**	14 (45.2)
**≥** **19**	17 (54.8)
**Sex**	
**Male**	18 (58.06)
**Female**	13 (41.94)
**Etiology**	
**Slip down**	16 (51.61)
**Traffic accident**	12 (38.71)
**Fall down**	1 (3.23)
**Collide**	1 (3.23)
**Assault**	1 (3.23)
**Non-surgical treatment method**	
**SAS**	23 (74.19)
**Arch bar**	4 (12.90)
**Circum-Mn wiring**	2 (6.45)
**Eyelet wiring**	1 (3.23)
**Facial band apply**	1 (3.23)
**Concomitant fracture**	
**No**	24 (77.42)
**Symphysis**	6 (19.35)
**Body**	1 (3.23)
**Fracture site**	
**Intracapsular fracture**	12 (38.7)
**Extracapsular fracture**	19 (61.3)
**Fractured segment location**	
**Inside glenoid fossa**	15 (48.39)
**Outside glenoid fossa**	16 (51.61)
**IMF duration (days)**	
**Mean (SD)**	17.52 (8.26)

Data are presented as percentages in parentheses

*SAS:* Skeletal anchorage system; *Circum-Mn wiring:* Circummandibular wiring; *IMF:* Intermaxillary fixation

### Methods

#### Data processing

**Fig. 2:**
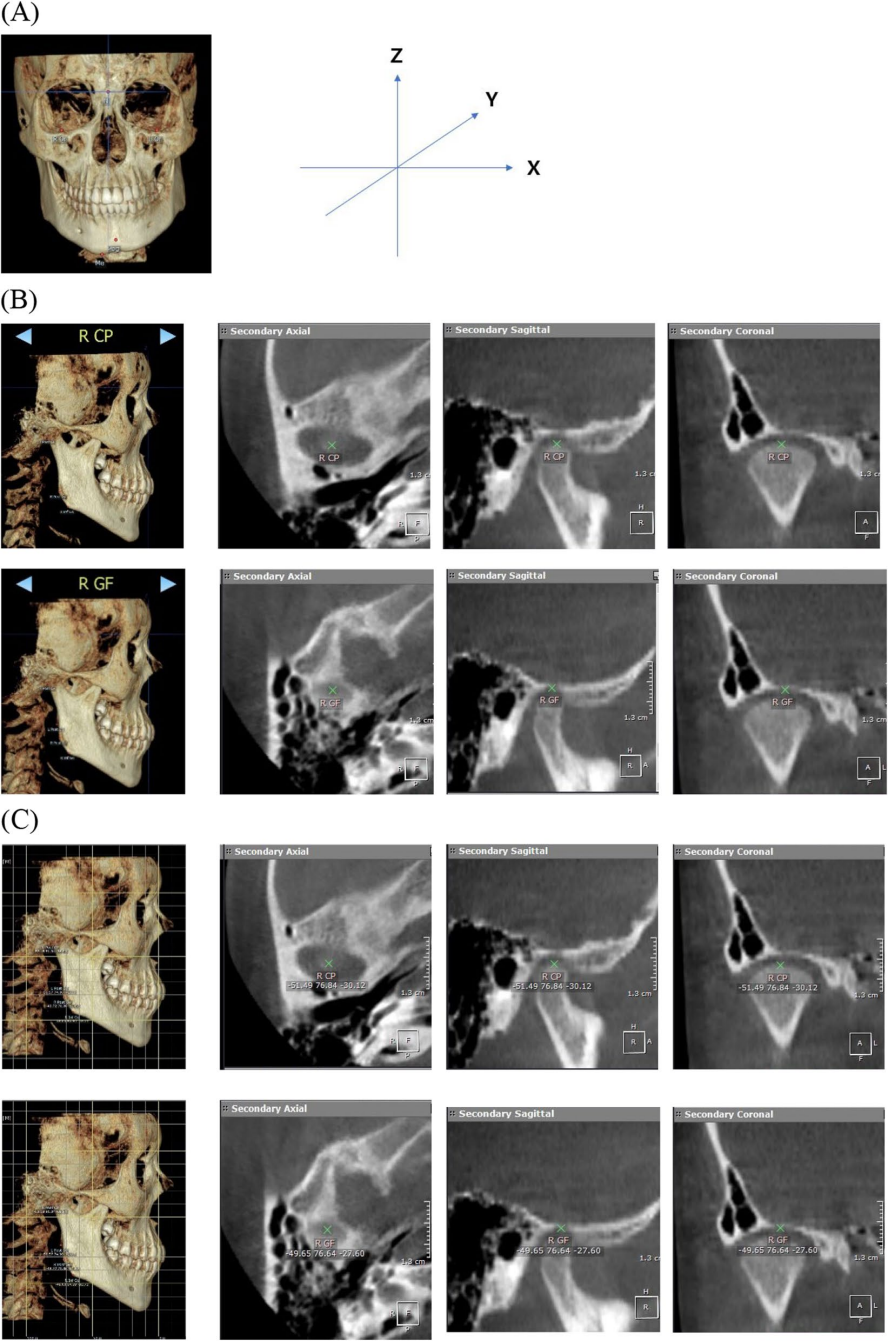
Data processing procedure. **A** Patients’ reconstructed 3D images were oriented based on the Frankfurt Horizontal (FH) plane method using Orbitale (Or) and Porion (Po). **B** On the axial, sagittal, and coronal sections of CT images, condylar point (CP) and glenoid fossa point (GF) of the affected and contralateral condyles were manually traced. **C** The *x*, *y*, and *z* coordinates of CP and GF of the affected and contralateral condyles were obtained

Digital Imaging and Communications in Medicine (DICOM) data were extracted and imported into OnDemand 3D App v.1.0 (CyberMed, Daejeon, Korea). After three-dimensional (3D) reconstruction, images were oriented based on the Frankfurt Horizontal (FH) plane method using orbitale (Or) and porion (Po). The *x*-, *y*-, and *z*-axes represent the medial–lateral, anterior–posterior, and superior-inferior positions, respectively. After defining the condylar point (CP) and glenoid fossa point (GF) on the OnDemand 3D application, the landmarks were manually traced on axial, sagittal, and coronal sections of CT images, and the *x*, *y*, and *z* coordinates were obtained (Fig. 2).

#### Measurement of condylar position

The patients underwent CT (Somatom Definition Edge, Siemens, Berlin, Germany) at the time of fracture (*T*_0_) and were treated non-surgically. CT images (Dinnova3, HDX, Seoul, Korea) were taken again after > 6 months of follow-up (*T*_1_). Three measurements of the two landmarks on the affected and contralateral condyles were obtained twice from CT images for all patients. The difference between the glenoid fossa and the condylar points was measured on the *x*-, *y*-, and *z*-axes. The landmarks and measurements used in this study are listed in Table 2.

**Table 2:** The landmarks and the measurements

	Definition
Landmarks	
Condylar point	Most superior point of mandibular condyle head
Glenoid fossa point	Most superior point of glenoid fossa
Measurements	
∆ GF-CP (*x*)	The difference between glenoid fossa point (*x*) and condylar point (*x*)
∆ GF-CP (*y*)	The difference between glenoid fossa point (*y*) and condylar point (*y*)
∆ GF-CP (*z*)	The difference between glenoid fossa point (*z*) and condylar point (*z*)

Three measurements of the two landmarks were obtained on the affected and contralateral condyles

*GF:* Glenoid fossa point; *CP:* Condylar point

#### Analyzing the amount of positional recovery

The extent of condylar position recovery along the *x*-, *y*-, and *z*-axes was measured by subtracting the distance from the GF to the CP between *T*_0_ and *T*_1_.

$$\text{Amount of recovery}\ (x) (\text{mm})=[\triangle\ \text{GF to CP}\ (x)]_\text{T0} - [\triangle\ \text{GF to CP }(x)]_\text{T1}$$

The difference between the GF and CP on the affected side was modified by removing that of the contralateral side. Unilateral condylar fractures made it possible to measure differences in the position between the fractured and contralateral sides, which offset the value produced by the articular disc and joint cavities between the temporal bone and condyle.

#### Clinical evaluation on prognosis

Clinical prognosis after non-surgical treatment was evaluated based on the incidence of complications. We reviewed the medical records of 31 patients who underwent clinical examination after > 6 months of remodeling. The indices included limitations on mouth opening, malocclusion, deviation of mouth opening, facial asymmetry, and TMD including pain, clicking, and crepitus.

#### Statistical analysis

Paired Student’s *t-*test was used to compare the positions of the condyle head at *T*_0_ and *T*_1_. Welch’s analysis of variance (ANOVA) was performed to confirm the difference in recovery along the *x*-, *y*-, and *z*-axes of the fractured mandibular condyle. Factors affecting recovery were evaluated using linear regression and Pearson’s correlation analysis. Intra-rater reliability was evaluated using the intra-class correlation coefficient (ICC) and Pearson’s correlation coefficient. The normality of the data was confirmed with an absolute skewness value < 2 and a kurtosis value < 7. Statistical analyses were performed using R software (v.4.3.0). *P-*values < 0.05 were considered statistically significant.

## Results

### Positional changes of mandibular condyle head

The average movement of the highest point of the condyle head at fractured state (*T*_0_) in 31 patients was 7.49 mm in the anterior direction, 4.78 mm in the inferior direction, and 4.70 mm in the medial direction. After > 6 months of remodeling (*T*_1_), the remodeled position of the condyle was 6.07 mm in the *y*-axis, 3.02 mm in the *z*-axis, and 2.96 mm in the *x*-axis. Positional differences between *T*_0_ and *T*_1_ on the *x*-, *y*-, and *z*-axes, and between the *x*-, *y*-, and *z*-axes at *T*_0_ and *T*_1_ were also statistically significant (*P* < 0.05) (Table 3).

**Table 3:** Positional changes on three-dimensional planes (unit: mm)

Variable	Fractured state (*T*_0_) Mean (SD)	> 6 months of remodeling (*T*_1_) Mean (SD)	*P-*value^1^
**∆ GF to CP (** ***x*** **)**	4.70 (4.39)	2.96 (4.56)	0.005^*^
**∆ GF to CP (** ***y*** **)**	7.49 (4.68)	6.07 (4.95)	0.028^*^
**∆ GF to CP (** ***z*** **)**	4.78 (2.27)	3.02 (2.49)	0.000^*^
***P*** **-value** ^**2**^	0.018^*^	0.011^*^	

Positional changes of condyle head among *x*-, *y*-, and *z*-axes and between *T*_0_ and *T*_1_ were statistically significant

*GF:* Glenoid fossa; *CP:* Condylar point; *T*_*0*_:The time of fracture; *T*_*1*_:After > 6 months of remodeling; *SD:* Standard deviation

^1^Statistical analyses are performed by paired *t*-test; ^*^: p < 0.05

^2^Statistical analyses are performed by Welch’s ANOVA; ^*^: p < 0.05

### Factor analysis for positional recovery

Univariate analysis was performed for six factors: sex, age, concomitant fracture, fracture site, intermaxillary (IMF) duration, and whether the fractured segment was located inside or outside the glenoid fossa. This analysis revealed that age is associated with the extent of recovery along the *x*- and *z*-axes. The location of the fractured segment inside or outside the glenoid fossa negatively affected the *x*-axis. Multivariate analysis of the identical factors revealed that being < 19 years old had a statistically significant positive influence on the recovery of position on the *x*- and *z*-axes (*P* < 0.05), whereas the fragment position inside the glenoid fossa was negatively associated with the amount of recovery on the *x*-axis (*P* < 0.05) (Table 4).

**Table 4:** Factor analysis for positional recovery of condylar fracture

Variable	Univariable (*x*)			Multivariable (*x*)			Univariable (*y*)			Multivariable (*y*)			Univariable (z)			Multivariable (*z*)		
	β	95% CI	*P*-value	β	95% CI	*P*-value	β	95% CI	*P*-value	β	95% CI	*P*-value	β	95% CI	*P*-value	β	95% CI	*P*-value
**Sex**																		
**Female (ref.)**	1			1			1			1			1			1		
**Male**	0.062	− 0.304 to 0.428	0.739	− 0.102	− 0.374 to 0.17	0.546	− 0.050	− 0.417 to 0.316	0.788	− 0.294	− 0.666 to 0.079	0.221	0.031	− 0.336 to 0.398	0.869	− 0.141	− 0.44 to 0.158	0.448
**Age**																		
**≥ 19 (ref.)**	1						1			1			1			1		
**<** **19**	0.566	0.316 to 0.816	0.001^*^	0.509	0.228 to 0.79	0.009^*^	0.215	− 0.135 to 0.565	0.345	0.127	− 0.27 to 0.523	0.608	0.591	0.352 to 0.83	0.000*	0.551	0.255 to 0.848	0.009*
**Concomitant fx**																		
**No** **(ref.)**	1						1			1			1			1		
**Symphysis**	0.096	− 0.27 to 0.462	0.615	− 0.105	− 0.43 to 0.22	0.602	− 0.083	− 0.448 to 0.282	0.664	− 0.003	− 0.456 to 0.451	0.992	− 0.077	− 0.044 to 0.29	0.688	− 0.234	− 0.591 to 0.124	0.296
**Body**	0.008	− 0.36 to 0.375	0.968	0.011	− 0.24 to 0.262	0.946	0.094	− 0.271 to 0.458	0.622	0.089	− 0.262 to 0.439	0.684	− 0.044	− 0.411 to 0.324	0.819	− 0.116	− 0.392 to 0.16	0.499
**Fracture site**																		
**Intracapsular** **(ref.)**	1			1			1			1			1			1		
**Extracapsular**	0.324	− 0.004 to 0.653	0.075	0.037	− 0.301 to 0.375	0.861	0.122	− 0.24 to 0.484	0.512	0.060	− 0.413 to 0.533	0.836	0.241	− 0.105 to 0.587	0.192	− 0.071	− 0.443 to 0.3	0.757
**Fractured segment position**																		
**Outside glenoid fossa** **(ref.)**	1			1			1			1			1			1		
**Inside glenoid fossa**	− 0.379	− 0.694 to − 0.065	0.035^*^	− 0.441	− 0.765 to − 0.117	0.035^*^	− 0.162	− 0.52 to 0.195	0.383	− 0.290	− 0.73 to 0.15	0.300	− 0.316	− 0.647 to 0.014	0.083	− 0.435	− 0.786 to − 0.084	0.056
**IMF duration**	− 0.278	− 0.617 to 0.062	0.131	− 0.325	− 0.618 to − 0.031	0.081	− 0.189	− 0.543 to 0.165	0.309	− 0.207	− 0.609 to 0.194	0.411	− 0.209	− 0.56 to 0.143	0.260	− 0.100	− 0.418 to 0.218	0.612

Univariable and multivariable analyses revealed that factors of age are influential on *x*- and *z*-axes; By linear regression; ^*^: p < 0.05

*< 19:* Under 19-year-old group; *≥ 19:* Over 19-year-old group; *fx:* Fracture; *IMF:* Intermaxillary fixation; *CI:* Confidence interval

### Age and extent of recovery

Pearson correlation coefficient (*r*) between age and recovery in the *x*- and *z*-axes were − 0.68 and − 0.62, respectively, indicating a statistically significant strong negative correlation (*P* < 0.001). However, the *y*-axis correlation coefficient was − 0.18 and was not statistically significant (*P* > 0.05) (Table 5). Average positional changes in the *x*- and *z*-axes were 4.18 and 2.86 between two age groups of under and over 19 years, which were statistically significant (*P* < 0.001) (Table 6).

**Table 5:** Correlation analysis between age and ∆ Fx-Rx (*x*,*y*,*z*)

Significance	∆ Fx-Rx (*x*)	∆ Fx-Rx (*y*)	∆ Fx-Rx (*z*)
**Pearson correlation coefficient (** ***r***)	− 0.68	− 0.18	− 0.62
***P-*** **value** ^1^	0.000^*^	0.328	0.000^*^

Pearson correlation coefficient (*r*) indicates strong negative correlations between age and recovery on the *x*- and *z*-axes, not on the *y*-axis. *∆ Fx-Rx (x, y, z)* difference of the position at *T*_0_ and *T*_1_on *x*-, *y*-, and *z*-axes respectively

^1^By Pearson’s correlation analysis; ^*^: p < 0.05

**Table 6:** Average positional changes between two age groups (unit: mm)

Variable	< 19 years (*n* = 14)	≥ 19 years (*n* = 17)	Mean differences	*P-*value^1^
**∆ Fx-Rx (** *x* **)**	3.74 (2.11)	− 0.43 (3.24)	4.18	0.000^*^
**∆ Fx-Rx (** ***y*** **)**	2.21 (4.44)	0.75 (2.17)	1.45	0.245
**∆ Fx-Rx (** ***z*** **)**	3.32 (2.11)	0.47 (1.91)	2.86	0.000^*^

Average positional changes in the *x*- and *z*-axes were statistically significant between the two age groups. *∆ Fx-Rx (x, y, z):* difference of the position at *T*_0_ and *T*_1_on *x*-, *y*-, and *z*-axes respectively

^1^By independent *t*-test; ^*^: p < 0.05

### Clinical prognosis of two age groups

In terms of clinical prognosis between the two age groups, there were two patients with temporomandibular joint disorders of crepitus and pain, and one suffered from malocclusion in the ≥ 19-year-old group. One pediatric patient aged 9 years was followed up for clinical asymmetry assessment in the < 19-year-old group. None of the patients in either group had mouth-opening limitations or deviations (Table 7).

**Table 7:** Clinical prognosis of patients between two age groups

Variable	< 19 (*n* = 14)	≥ 19 (*n* = 17)	Total (*n* = 31)
**MOL**			
**No**	14	17	31
**Yes**	0	0	0 (0.0%)
**Occlusion**			
**Favorable**	14	16	30
**Unfavorable**	0	1	1 (3.2%)
**TMD**			
**No symptom**	14	15	29
**Yes (pain, crepitus, click)**	0	2	2 (6.4%)
**Deviation on opening**			
**No**	14	17	31
**Yes**	0	0	0 (0.0%)
**Facial asymmetry**			
**No**	13	17	30
**Yes**	1	0	1 (3.2%)

All complications except for facial asymmetry have arisen in ≥ 19-year-old group

*<19:* Under 19-year-old group; *≥* *19:* over 19-year-old group; *MOL:* Mouth opening limitation; *TMD:* Temporomandibular joint disorder

### Intra-rater reliability

The 31 sets of data along the *x*-, *y*-, and *z*-axes were obtained twice. Sixteen datasets (50% of all subjects) were randomly selected, and the intra-rater reliability was evaluated using the intra-class correlation coefficient (ICC), with a confidence interval (CI) of 95%. Pearson correlation coefficient (*r*) was used to perform the correlation analysis. ICC values were distributed from 0.85 to 1.00 and Pearson’s *r* values were in a range from 0.84 to 1.00, which indicated almost perfect agreement (Table 8).

**Table 8:** Intra-rater reliability

	At the fractured state (*T*_0_)						After > 6 months of remodeling (*T*_1_)					
	Affected side			Contralateral side			Affected side			Contralateral side		
	*x*	*y*	*z*	*x*	*y*	*z*	*x*	*y*	*z*	*x*	*y*	*z*
**ICC**	1.00	0.99	0.97	0.85	0.99	0.98	0.96	0.96	0.94	0.94	0.86	0.98
**95% CI**	0.99–1.00	0.98–1.00	0.92–0.99	0.62–0.94	0.99–1.00	0.94–0.99	0.88–0.98	0.89–0.99	0.83–0.98	0.82–0.98	0.64–0.95	0.94–0.99
***r***	1.00	0.99	0.97	0.84	0.99	0.98	0.97	0.96	0.94	0.94	0.87	0.98

Randomly selected sixteen datasets showed almost perfect agreement

*ICC:* Intra-class correlation coefficient; *CI:* Confidence interval; *r:* Pearson correlation coefficient

## Discussion

Mandibular condylar fractures can be treated with open or closed reduction, which has been controversial for decades. With closed reduction, positional changes and remodeling of the condyle are questionable compared with open reduction. In the fractured condyle, the proximal fragment is presumed to move under the action of the lateral pterygoid muscle. The displaced bone fragment moves anteromedially in the direction that is pulled toward the pterygoid plate, the origin of the lateral pterygoid. In terms of direction, one group of researchers reported that medial translation was statistically significant in closed treatment after overlapping the right and left condyles in 21 patients [5]. In this study, the extent of movement and direction of the fractured mandibular condyle were evaluated. We found that the fractured bone segments recovered the position, on average, after non-surgical treatment. Specifically, the fractured condyles moved medially on the *x*-axis, anteriorly on the *y*-axis, and inferiorly on the *z*-axis. The *x*-, *y*-, and *z*-axes were in the order of anterior > inferior > medial directions at *T*_0_. This was the same after > 6 months of bone remodeling. The difference between the timepoints was statistically significant, which indicates that the position of the condyle head recovered.

Changes in condylar position and traumatic force can influence surrounding anatomical structures. Adjacent structures such as the articular disc and retrodiscal tissue can be damaged or moved together with a displaced segment. Certain studies that analyzed the soft tissue around the injured temporomandibular joint in 18 patients using Magnetic Resonance Imaging (MRI) revealed 15 anteriorly and inferiorly displaced discs, 9 torn capsules, 16 torn retrodiscal tissue events, and 19 cases of joint effusion [6]. Another study reported that retrodiscal tissue tearing was observed in 74.4% of the MRI of 129 patients with intracapsular condylar fractures [7]. In 20 pediatric patients with sagittal condylar fractures, researchers found that 77.4% of articular discs were displaced anteriorly. Anteriorly sustained 19.3% of discs after closed reduction resulted in unfavorable remodeling [8]. In the present study, we found that the condyle head that moved in the anterior direction recovered less in the posterior direction than in the lateral or superior directions. We also confirmed that the extent of posterior recovery on the y-axis did not significantly correlate with age. We speculated that, as the proximal fragment was moved forward by the lateral pterygoid, the retrodiscal tissue or capsule was torn or damaged histologically. This impairment could make it difficult to reduce the proximal segment posteriorly, which needs to be considered in the treatment of condylar fractures, even in pediatric patients.

There have been several arguments for non-surgical treatment, especially in pediatric patients, with few complications and favorable remodeling after closed reduction. Ghasemzadeh and colleagues [9] reviewed the medical records of 43 patients and reported that a closed reduction in pediatric patients resulted in few complications. In terms of bone remodeling after closed treatment, some studies or cases have indicated favorable outcomes [10–13]. Lindahl et al. [14] studied the radiographs of 76 patients, who were divided into four age groups: 3–11, 12–15, 16–19, and > 20 years. The condyles were completely remodeled to normal articulation in 20 of 27 children, recovered to a normal extent in teenagers, and only minor remodeling was observed in adults. They divided restitutional and functional remodeling into pediatric and adult patients, respectively. Ellis and Throckmorton [15] explained why the pattern of remodeling differs according to patient age, as condylar cartilage hypertrophy is possible at a younger age. In this study, we considered that various factors may be involved in the positional recovery of the fractured mandibular condyle. The independent variables included were sex, age, concomitant fracture, fracture site, IMF duration, and location of the fractured segment (inside or outside the glenoid fossa). Univariate and multivariate analyses of the aforementioned factors revealed that patient age had a statistically significant influence on lateral and superior recovery. Additionally, condylar positional change exhibited a negative correlation with age only in terms of lateral and superior recovery, but not in posterior movement. While a positive recovery in position was observed in the < 19-year-old group, there was little change in the ≥ 19-year-old group. This age-related recovery leads us to several speculations on the reasons including the attachment status of the lateral pterygoid muscle, occlusion, mandibular morphology, and possibility of compensatory growth of condyle, which could be clarified in further research.

In several studies regarding restitutional remodeling of condyles in pediatric patients, the relationship between the fractured condyle and the glenoid fossa at *T*_0_ has been speculated on. A group of researchers elucidated that stress stimulation inside the glenoid fossa is a prerequisite for condylar remodeling after reviewing 27 children with extracapsular fractures [16]. In this study, we performed multivariate analysis and found that the functional force inside the glenoid fossa did not have a positive influence on the extent of recovery of fractured condyles. Further research involving larger sample sizes is recommended to confirm these results.

Functional prognosis is an important aspect in the evaluation of treatment results because favorable functional recovery without anatomical reduction after non-surgical treatment of condylar fractures is uncertain. Marker et al. [17] analyzed the results of conservative treatment in 348 patients with mandibular condylar fractures and reported that 13% of them had physical complaints such as mouth opening limitation and deviation on opening. TMD and malocclusion occurred in 3% and 2% of patients, respectively. The authors concluded that closed reduction is a safe and predictable method. Lee et al. [18] reported that the incidence of clinical complications was not significantly different between open and closed reductions in 198 patients. On the other hand, there was a study that unfavorable functional prognosis showed depending on the types of condylar fracture [19]. In the present study, functional prognosis was observed clinically after non-surgical treatment in patients with unilateral condylar fractures, and treatment results were favorable in most patients. We divided patients into two groups according to age, which was the only influential factor in positional recovery. There was a low percentage of functional complications of TMD and malocclusion only in the ≥ 19-year-old group and one case of facial asymmetry was observed in the < 19-year-old group. The only facial asymmetry occurred in the 9-year-old patient with a severely deviated extracapsular fracture outside the glenoid fossa. The amount of segment deviation was measured to be 11.24, 4.74, and 3.93 mm in *x*-, *y*-, and *z*-axes respectively. The asymmetry was corrected with orthognathic surgery at the age of 25. When non-surgical treatment is applied, different complications should be considered and informed to the patients depending on their age.

This study has certain limitations. First, it was designed retrospectively. Second, this study included patients with unilateral condylar fractures. It cannot be applied equally to patients with bilateral condylar fractures. Third, more significant results could be obtained with larger sample sizes.

## Conclusion

In mandibular condylar fractures, the condyle head moves anteromedially and inferiorly at the time of the fracture. After non-surgical treatment, the condyle head significantly recovered laterally and superiorly in the < 19-year-old age group, and the functional prognosis was favorable in all age groups. Non-surgical treatment can be an applicable treatment option for patients with mandibular condylar fractures.

## Data Availability

No datasets were generated or analysed during the current study.

## Abbreviations

TMD: Temporomandibular disorder
TMJ: Temporomandibular joint
CT: Computed tomography
SAS: Skeletal anchorage system
Circum-Mn: Circummandibular
IMF: Intermaxillary fixation
DICOM: Digital imaging and communications in medicine
3D: Three-dimensional
FH: Frankfurt horizontal plane
Or: Orbitale
Po: Porion
CP: Condylar point
GF: Glenoid fossa point
Fx: Fracture
Rx: Remodeling
